# Angiotensin receptor blocker and angiotensin-converting enzyme inhibitor use and survival in gastric cancer patients: a Finnish nationwide cohort study

**DOI:** 10.1007/s10120-025-01662-2

**Published:** 2025-09-20

**Authors:** Aliisa Auvinen, Panu Aaltonen, Harri Mustonen, Caj Haglund, Pauli Puolakkainen, Hanna Seppänen

**Affiliations:** Department of Surgery, Translational Cancer Medicine Research Program, iCAN Digital Precision Cancer Medicine Flagship, Faculty of Medicine, University of Helsinki and Helsinki University Hospital, Haartmaninkatu 8, PL 63, 00014 Helsinki, Finland

**Keywords:** Gastric cancer, Survival, Epidemiology, Angiotensin receptor blocker, Angiotensin-converting enzyme inhibitor

## Abstract

**Background:**

The renin–angiotensin system (RAS) has been increasingly recognized to be associated with carcinogenesis and cancer progression. There is extensive preclinical evidence suggesting the benefits of RAS-inhibiting drugs, such as angiotensin receptor blockers (ARBs) and angiotensin-converting enzyme inhibitors (ACEIs), in preventing the progression of gastric cancer (GC). However, clinical evidence supporting the positive effects of ARBs and ACEIs on GC prognosis is currently limited. The purpose of this study is to investigate their effects in a Finnish cohort.

**Methods:**

This is a retrospective national cohort study, where cancer patient registry data were linked to prescription purchase records for ARBs and ACEIs. The effect of ARB/ACEI in the post-diagnostic period on overall mortality was assessed using Cox regression analysis. Disease-specific mortality associations were evaluated with the Fine and Gray model.

**Results:**

We included 2246 histologically confirmed GC patients diagnosed between 2011 and 2016. Follow-up continued until the end of 2023. In the main analysis, a protective effect of ARB use was associated with a significant reduction in overall mortality (adjusted hazard ratio (HR) 0.81, 95% confidence interval (CI) 0.69–0.94, *p* = 0.007). Furthermore, the effect was greater for those with higher ARB dosage. A similar finding was not observed with ACEI use. For disease-specific survival, both ARB and ACEI use had a significant protective effect (adjusted HR 0.75, 95% CI 0.62–0.90 *p* = 0.002 and adjusted HR 0.76, 95% CI 0.63–0.93, *P* = 0.007, respectively).

**Conclusions:**

Our study adds to the evidence that ARB use might have a beneficial impact on survival among GC patients.

**Supplementary Information:**

The online version contains supplementary material available at 10.1007/s10120-025-01662-2.

## Introduction

Gastric cancer (GC) ranks among the top 10 cancers worldwide in both incidence and cancer-related death rates [1]. It is often diagnosed at advanced stages and the prognosis remains poor even in developed countries [2]. Therefore, it is crucial to find agents that can improve GC survival.

Angiotensin receptor blockers (ARBs) and angiotensin-converting enzyme inhibitors (ACEIs) are widely used drugs for hypertension and heart failure. They inhibit the renin–angiotensin system (RAS), which has traditionally been recognized as a regulator of blood pressure and cardiovascular homeostasis. However, it is now known that local RASs are found in various organs and tissues, mediating diverse physiological functions [3]. In particular, angiotensin II (ATII), via angiotensin II type 1 (AT1) receptors, directly affects cell proliferation, angiogenesis, and inflammation in many tissues [3]. Local RAS components are also found in different neoplasms, and RAS upregulation is often correlated with tumor progression, vascularization, and metastasis in preclinical studies [4, 5].

For GC specifically, preclinical studies have demonstrated that AT1 receptors are overexpressed in malignant gastric tissue compared to non-lesion tissue [6, 7]. AT1 receptors, among other RAS components, are associated with angiogenesis, proliferation and lymph node spread in GC cell lines, rodent models, and tissue samples [6–9]. Conversely, blocking AT1 receptors inhibits these functions [6, 7, 9–12]. Moreover, RAS component expression is found to be higher in the gastric mucosa of patients with *Helicobacter pylori* (*H. pylori*) infection, a well-known risk factor for GC [13]. In rodent models, the upregulation of RAS increases as *H. pylori* infection and gastric inflammation progress [14]. RAS is also linked to stress-induced gastric ulceration in rodent models [15, 16]. These findings suggest that RAS plays a significant role in different gastric morbidities, lesions, and injuries.

While the preclinical evidence is extensive, clinical studies on ARBs/ACEIs and GC remain scarce. The association between ARB or ACEI use and improved survival among GC patients is based on a limited number of studies. Moreover, only one study involves a Western cohort, while the others are from Asian cohorts. For GC, this distinction might be necessary as several differences in characteristics of the disease have been described between Asian and Western populations, including treatment outcomes [17]. This study investigates the association between ARB and ACEI use and survival outcomes among GC patients in the Finnish population, thereby contributing to the Western perspective on the disease.

## Patients and methods

### Data

Patients diagnosed with GC (ICD-10 code C16) between 2000 and 2016 were identified from the Finnish Cancer Registry (FCR). These patients are also described in a previous publication [18]. The FCR records all cancer cases in Finland through legislation-enforced reporting. The FCR data were linked to patients’ healthcare visits from the National Institute for Health and Welfare’s Care Register for Health Care (HILMO) and the Register of Primary Health Care Visits (avoHILMO). Treatment modalities (surgery, radiotherapy, and chemotherapy) were identified using the Nordic Medico-Statistical Committee Classification of Surgical Procedures [19]. For patients treated in the Helsinki and Uusimaa Hospital District, additional oncological treatment data were collected from local registries and compared and combined with HILMO data. Data on the time and cause of death and residence were obtained from Statistics Finland, with follow-up until the end of 2023.

Data on ARB and ACEI purchases were obtained from the Finnish Social Insurance Institution (SII), which maintains a national prescription purchase database for all Finnish citizens. ARB/ACEI purchases were identified by Anatomical Therapeutic Chemical (ATC) codes and linked to patient data through a unique identification number. Additionally, the data included date of purchase, dose, package size, and number of packages purchased.

### Study design and population

This national register-based cohort study included patients with histologically confirmed adenocarcinoma. Exclusion criteria were age under 18 (*n* = 3), diagnosis based on a death certificate (*n* = 116), an autopsy (*n* = 885), or a clinical examination with no histology (*n* = 11), and other histologies including neuroendocrine (*n* = 556), gastrointestinal stromal tumors (*n* = 139), unspecified neoplasms (*n* = 496), mesenchymal tumors, as well as other tumors with rare histologies (*n* = 81). Patients diagnosed before 2011 (*n* = 6200) were excluded as the prescription purchase data were only available from 2011. Finally, those with both ARB and ACEI use during the follow-up period (*n* = 18), with missing information on university catchment area (*n* = 19) and who died shortly (within 91 days) after diagnosis (*n* = 731) were excluded from the cohort.

### Drug categories and definition of use

In our analyses, post-diagnostic ARB or ACEI use was modeled. All ARBs and ACEIs used by the cohort were considered, and a full list of them is provided in Supplement Table S1.

Patients were classified as users upon purchasing the equivalent of three months (91 days) of cumulative use in the post-diagnostic period, determined by the defined daily dose (DDD) system. Before reaching this threshold, patients were in the non-user group to avoid immortal time bias. The DDD is a measure of drug consumption defined by the World Health Organization that allows comparison between different drugs [20]. Drug quantities in milligrams were calculated per purchase in the post-diagnostic period, divided by DDD, and then a running cumulative amount of DDD (cDDD) was calculated for every patient. Missing data on dose or amount purchased (0.7% of records) were imputed based on other purchases by the patient or, if missing, the most common values were assumed. Once patients entered the user category, they remained there for the rest of the study period. This approach was taken to control for bias arising from selective discontinuation of preventive drugs during palliative care [21].

The exposed patients were further categorized as low-dosage and high-dosage users based on the median amount of cDDD of ARB/ACEI in this group. Patients entered the low-dosage group upon becoming users and further entered the high-dosage group upon reaching the median cDDD, in a time-dependent manner.

### Outcome measurements and covariates

The primary outcome was overall survival (OS) from diagnosis. Covariates included age, sex, comorbidities, resection, oncological treatments, and university hospital area. We did not use stage information as the FCR database does not use TNM staging, and the accuracy of its stage data has previously been evaluated as limited [22, 23]. In addition, stage data were missing for majority of patients included in our study. Resection was defined as either a total or a partial gastrectomy, performed as either open or minimally invasive surgery. The non-resection group included patients who intended to undergo radical surgery but were found to be non-resectable during operation. Oncological treatment information included chemotherapy and radiotherapy. Information on treatment intent (curative, downstaging, or palliative) was not available. Comorbidities were evaluated using the Charlson comorbidity index (CCI) [24–26] based on healthcare visits preceding GC diagnosis during the period from 2000 to 2016.

### Statistical analysis

Descriptive statistics were calculated for the overall cohort as well as for patients with post-diagnostic ARB/ACEI use and for the non-user group. Demographic differences were compared with Pearson’s chi-squared test and Kruskal–Wallis rank-sum test for categorical and continuous variables, respectively. Simon–Makuch plots, an extension of the Kaplan–Meier that accounts for time-dependent variables, were generated to visually compare the survival curves [27].

The Cox proportional hazards model was used to calculate hazard ratios (HRs) and adjusted HRs for OS. Time was defined as the number of days from GC diagnosis, with follow-up continuing until death or censoring on the common closing date of December 31, 2023. ARB/ACEI use, resection status, and oncological treatment were included as time-dependent covariates to address immortal time bias. The proportional hazard (PH) assumption was evaluated visually with Schoenfeld residual plot and testing for a trend. The multivariate Cox model included age, sex, CCI score, resection, and any oncological treatment. Because of violations of the PH assumption, surgery and oncological treatment were added as stratified variables. Additionally, the model was stratified by the university catchment area to account for group variation.

Subgroup analyses were done based on resection status. In addition, the ARB/ACEI use was analyzed by dosage group to assess dose–response relationship. As a sensitivity analysis, the Fine and Gray model was used to conduct a competing risk analysis for disease-specific survival (DSS).

All analyses were performed using R (4.3.1; Foundation for Statistical Computing, Vienna, Austria). All *p *values reported are two-sided, and *p* < 0.05 was considered statistically significant. The study protocol was approved by the National Institute for Health and Welfare, Statistics Finland, and the Helsinki University Hospital.

## Results

A cohort of 2246 histologically confirmed GC patients was included in the study. Among these participants, 282 (12.6%) were ARB users and 266 (11.8%) were ACEI users, each with more than 3 months of cumulative cDDD. By the end of the follow-up period, 1904 (84.8%) of the cohort had died, including 185 (65.6%) patients in the ARB group and 202 (75.9%) in the ACEI group. The median follow-up time was 16.6 months (interquartile range 7.9–54.0 months). Baseline characteristics for the overall cohort and by drug use are presented in Table 1. ARB and ACEI users had a higher frequency of gastric surgeries and were slightly older at diagnosis. ACEI users had higher number of comorbidities compared to non-users and ARB users. Specifically, ACEI users had higher rates of myocardial infarctions, congestive heart failure, peripheral vascular disease, cerebrovascular disease, and dementia compared to non-users. In addition, both ARB and ACEI users had more diabetes without chronic complications compared to non-users (Supplement Table S2).

**Table 1:** Demographic and clinical characteristics

	Overall		Non-user		ARB user		ACEI user		
	*n*	(%)	*n*	(%)	*N*	(%)	*n*	(%)	*p*^a^
Total	2246		1698		282		266		
Age at diagnosis	69.5		69.0		70.5		71.7		0.009
Sex									0.028
Male	1343	(60)	1018	(60)	152	(54)	173	(65)	
Female	903	(40)	680	(40)	130	(46)		()	
CCI									< 0.001
CCI 0	1000	(45)	801	(47)	112	(40)	87	(33)	
CCI 1	500	(22)	370	(22)	65	(23)	65	(24)	
CCI 2	364	(16)	262	(15)	53	(19)	49	(18)	
CCI 3 +	382	(17)	265	(16)	52	(18)	65	(24)	
Oncological treatment^b^									0.7
No	1293	(58)	971	(57)	163	(58)	159	(60)	
Yes	953	(42)	727	(43)	119	(42)	107	(40)	
Resection									< 0.001
No	1326	(59)	1061	(62)	128	(45)	137	(52)	
Yes	920	(41)	637	(38)	154	(55)	129	(48)	
Stage									< 0.001
Local	157	(16)	97	(13)	27	(25)	33	(32)	
Locally advanced	288	(29)	214	(28)	39	(36)	35	(34)	
Metastatic	537	(55)	461	(60)	41	(38)	35	(34)	
Unknown	1264		926		175		163		
Histology^c^									0.005
Intestinal	585	(43)	420	(41)	85	(49)	80	(52)	
Diffuse	774	(57)	614	(59)	87	(51)	73	(48)	
Unknown	887		664		110		113		
Tumor location									0.7
Cardia	504	(38)	373	(38)	60	(37)	71	(41)	
Non-cardia	823	(62)	616	(62)	103	(63)	104	(59)	
Unknown	919		709		119		91		
University hospital area									> 0.9
Helsinki	620	(28)	460	(27)	80	(28)	80	(30)	
Kuopio	389	(17)	292	(17)	54	(19)	43	(16)	
Oulu	331	(15)	252	(15)	39	(14)	40	(15)	
Tampere	492	(22)	373	(22)	61	(22)	58	(22)	
Turku	414	(18)	321	(19)	48	(17)	45	(17)	

^q^Kruskal–Wallis rank-sum test, Pearson’s chi-squared test

^b^Chemotherapy and/or radiotherapy

^c^By Lauren et al. [28]

*ARB* angiotensin receptor blocker, *ACEI* angiotensin-converting enzyme inhibitor, *CCI* Charlson comorbidity index

**Fig. 1:**
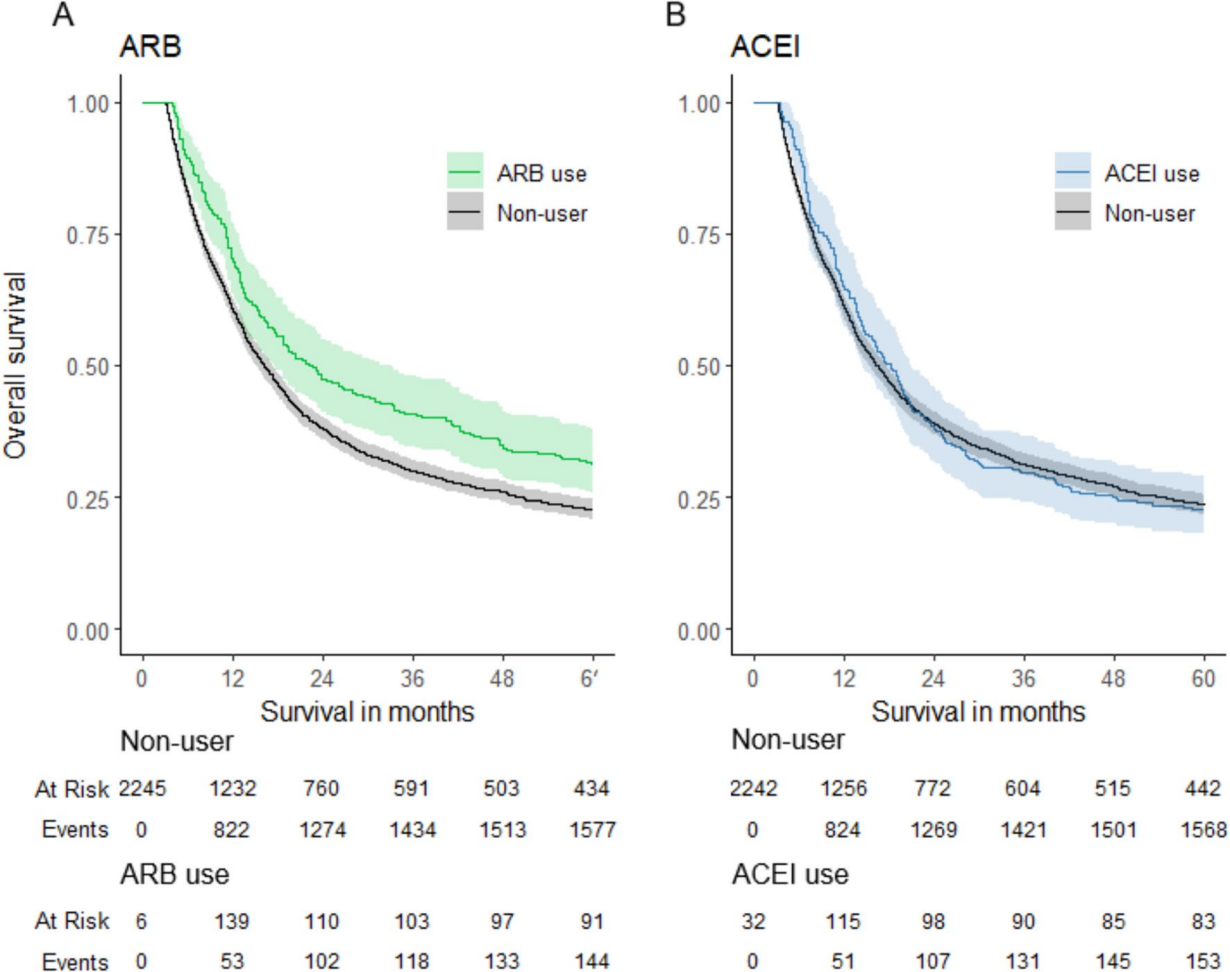
Simon–Makuch plots for overall survival of gastric cancer patients by **a** ARB users versus non-users and **b** ACEI users versus non-users. *ARB* angiotensin receptor blocker, *ACEI* angiotensin-converting enzyme inhibitor

In the main analysis, ARB showed a statistically significant protective effect, while ACEI did not. Age and CCI score of 3 or more were associated with a slight reduction in OS, but sex and lower CCI scores did not have a significant effect. The results of the main analysis are detailed in Table 2. Figure 1 presents Simon–Makuch plots of ARB and ACEI use versus overall survival.

**Table 2:** Association of ARB and ACEI use and mortality among gastric cancer patients

		Univariate			Multivariate^a^		
		HR	(95%) CI	*p*	HR	(95% CI)	*p*
ARB^b^		0.81	(0.69–0.94)	0.007	0.77	(0.66–0.90)	0.001
ACEI^b^		1.11	(0.96–1.29)	0.2	0.97	(0.83–1.13)	0.7
Age		1.02	(1.01–1.02)	< 0.001	1.02	(1.01–1.02)	< 0.001
Sex							
	Female	Ref			Ref		
	Male	1.01	(0.92–1.11)	0.8	0.96	(0.88–1.06)	0.4
CCI							
	0	Ref			Ref		
	1	1.00	(0.89–1.13)	> 0.9	1.00	(0.89–1.13)	> 0.9
	2	1.13	(0.99–1.28)	0.071	1.09	(0.96–1.25)	0.2
	3 +	1.26	(1.11–1.43)	< 0.001	1.17	(1.02–1.33)	0.023

^a^Adjusted by age, sex, Charlson comorbidity index and stratified by surgery, oncological treatment and university catchment area

^b^Use defined by at least 91 cumulative defined daily dose of drug use in the post-diagnostic period

*HR* hazard ratio, *CI* confidence interval, *ARB* angiotensin receptor blocker, *ACEI* angiotensin-converting enzyme inhibitor, *CCI* Charlson comorbidity index

For DSS, both ARB and ACEI use were associated with a statistically significant protective effect of similar magnitude. In subgroup analysis by resection status, ARB use was linked to improved survival among GC patients without resection but not in those who underwent surgery; however, the interaction between ARB and resection status was not statistically significant (*p* for interaction = 0.056). ACEI use did not have a significant effect in either subgroup. A dose–response relationship was observed for ARB, with patients in the high-dosage group exhibiting lower hazard ratios compared to those in the low-dosage group. These results are presented in Table 3.

**Table 3:** Association of ARB and ACEI use and mortality among gastric cancer patients in a competing risk analysis, by surgery and by dosage group

Drug/analysis	ARB						ACEI					
	Univariate			Multivariate^a^			Univariate			Multivariate^a^		
	HR	(95% CI)	*p*	HR	(95% CI)	*p*	HR	(95% CI)	*p*	HR	(95% CI)	*p*
Main analysis	0.81	(0.69–0.94)	0.007	0.77	(0.66–0.90)	0.001	1.11	(0.96–1.29)	0.159	0.97	(0.83–1.13)	0.672
DSS^b^	0.78	(0.65–0.94)	0.008	0.75	(0.62–0.90)	0.002	0.79	(0.65–0.96)	0.019	0.76	(0.63–0.93)	0.007
Surgery^c^												
No resection	0.71	(0.57–0.87)	0.001	0.69	(0.56–0.85)	< 0.001	1.03	(0.85–1.25)	0.762	0.94	(0.77–1.14)	0.513
Resection	0.94	(0.75–1.19)	0.619	0.89	(0.70–1.13)	0.346	1.14	(0.90–1.43)	0.271	1.02	(0.80–1.30)	0.861
Dosage group^d^												
Low-dosage	0.83	(0.69–1.00)	0.056	0.82	(0.68–0.99)	0.042	0.97	(0.78–1.21)	0.8	1.08	(0.89–1.31)	0.4
High-dosage	0.76	(0.59–0.99)	0.040	0.67	(0.52–0.87)	0.003	0.71	(0.47–1.05)	0.088	0.83	(0.65–1.04)	0.11

^a^Adjusted by age, sex, Charlson comorbidity index and stratified by surgery, oncological treatment and university catchment area

^b^Competing risk analysis against non-gastric cancer death using the Fine and Gray model

^c^Interaction *p* = 0.086 for ARB and surgery, interaction *p* = 0.5 for ACEI and surgery

^d^Dosage group defined by the median cumulative defined daily dose (cDDD) in the post-diagnostic period. Median cDDD were 588 for ARB and 596 for ACEI

*HR* hazard ratio, *CI* confidence interval, *ARB* angiotensin receptor blocker, *ACEI* angiotensin-converting enzyme inhibitor, *DSS* disease-specific survival

## Discussion

In our analyses, ARB use was consistently associated with improved survival in GC patients, whereas ACEI use did not demonstrate a significant effect in the main analysis. Additionally, evidence for a dose–response effect was observed for ARBs, where dosages higher than the median were associated with reduced mortality compared to lower dosages. Both ARBs and ACEIs were linked to a similar protective association for disease-specific survival.

We found that ARB use was associated with improved survival in GC patients. To date, three cohort studies have assessed the impact of ARB and ACEI use on survival among GC patients [29–31]. Busby et al. reported a moderate reduction in gastro-esophageal cancer mortality among ARB users in an English cohort of 2733 patients [29], while Cui et al. found a similar association in a Shanghai cohort of 544 patients 29). Li et al. observed a survival benefit for ARB/ACEI users in a Taiwanese cohort of 3254 but did not analyze the drug classes separately [31]. Consistent with our findings, both Busby et al. and Cui et al. reported evidence of a dose–response relationship using cDDDs for ARB and for ARB/ACEI use, respectively. However, Li et al. did not assess dose–response effects.

In contrast to ARBs, ACEIs effect on survival was less clear. In the previous studies, Busby et al. and Cui et al. did not find evidence of protective effect for ACEIs among GC patients. In fact, Busby et al. used ACEI users as a negative control group for their analysis. Since Li et al. combined ARB and ACEI use, the independent effects of these drug classes could not be determined. It has been speculated that ARBs may demonstrate better clinical outcomes than ACEIs due to their ability to preserve angiotensin [1–7] levels, a peptide associated with the reverse effects of Ang II [32]. In contrast, ACEIs reduce the production of angiotensin and accumulate bradykinin, which may potentially offset the benefits of RAS blockade [32].

We found that the protective effect of ARBs was more pronounced in the non-resection group, while no clear benefit was observed among patients who underwent resection. In their findings, Busby et al. and Li et al. reported similar effect sizes for operated GC patients compared to the overall cohort. As the interaction between ARBs and resection status was not significant, it is possible the observed subgroup difference reflects random variation in our data. Due to the FCR data being largely incomplete for stage, Lauren classification, and tumor location (56.3%, 39.5% and 40.9% of records were unknown, respectively), we could not examine these groups more closely. Li et al. reported a stronger protective effect of ARB/ACEI use in patients with lower-stage GC. [31]. Tumor type might play a role in the association between ARB/ACEI use and survival. A preclinical study by Röcken et al. investigated tissue samples from GC patients and found that ATR1 receptors are significantly more prevalent in intestinal-type cancer than in diffuse-type cancer [33]. In fact, none of the diffuse-type cancers included in the study were AT1 receptor positive. Tumor location may also be relevant as *H.* *pylori* infection plays a clearer role in non-cardia cancers compared to cardia cancers [34], making non-cardia cancers more promising targets for RAS blockade when considering the role of *H. pylori* in RAS upregulation discussed in the Introduction. Furthermore, cardia cancers are thought to have heterogenous etiologies, where some tumors are associated with a similar pathogenesis to that of lower esophageal adenocarcinoma [35]. Prior studies have not demonstrated survival benefits from ARB/ACEI use for esophageal cancers; however, none of them report HRs for esophageal adenocarcinoma specifically [29, 31, 36]. Additionally, it should be noted that the study by Röcken et al. did not find a difference in the prevalence of AT1 receptors by the location of GC (gastro-esophageal junction, body/fundus, or antrum/pylorus).

Our study utilized comprehensive registers: FCR, HILMO, and the SII database, ensuring that our dataset had excellent coverage of the Finnish population. The completeness and accuracy of FCR and HILMO have been previously evaluated as good [37–40]. In Finland, ARBs or ACEIs are not available over-the-counter and all prescription purchases of these medications are reimbursed by the SII. This structure provides high coverage for our prescription purchase data, eliminating recall bias and minimizing exposure misclassification due to over-the-counter use. Furthermore, our study provides evidence of a dose–response relationship for ARB use, which mitigates bias from reverse causation.

Nevertheless, our study has several limitations. As an observational study, it is subject to confounding due to incomplete or unmeasured covariates. We were unable to estimate pre-diagnostic use for those diagnosed around the start of the study period. Patients who underwent GC surgeries included both radical and palliative operations. While it is expected that the resection group would have a greater proportion of lower-stage cancers, the resection status does not completely represent the cancer stage. In relation to this, the incomplete stage data might be a concerning limitation since ARB and ACEI users had more resections and therefore might have better survival due to lower cancer stages compared to non-users. This study may also be subject to the healthy user bias. These concerns are mitigated to some extent by the fact that only ARBs demonstrated a clear effect on overall survival for GC patients since ARBs and ACEIs have similar indications. However, this interpretation is complicated by the higher comorbidity burden among ACEI users even though we did adjust the analysis by CCI. Moreover, bias by indication cannot be fully excluded, particularly given the lack of information on the specific indications for which ARBs/ACEIs were prescribed.

## Conclusions

This nationwide cohort study demonstrates that ARB use is beneficial among GC patients, particularly for those who did not receive surgical treatment. Conversely, for ACEIs, the reduction of mortality was observed for cancer-specific mortality but not for overall mortality. Future research should focus on investigating the effects of RAS blockade at different stages of GC, comparing intestinal-type cancer to diffuse-type cancer, as well as exploring its implications in esophageal adenocarcinomas.

## Supplementary Information

Below is the link to the electronic supplementary material.

Supplementary file1 (PDF 222 kb)

## Data Availability

Due to legal restrictions, the data underlying this study cannot be shared.

## Abbreviations

RAS: Renin–angiotensin system
ARB: Angiotensin receptor blocker
ACEI: Angiotensin-converting enzyme
GC: Gastric cancer
ATII: Angiotensin II
AT1: Angiotensin II type 1
*H*. *pylori*: *Helicobacter* *pylori*
FCR: Finnish cancer registry
HILMO: Care register for health care
ATC: Anatomical therapeutic chemical
DDD: Defined daily dose
cDDD: Cumulative amount of DDD
OS: Overall survival
CCI: Charlson comorbidity index
HR: Hazard ratio
PH: Proportional hazard
DSS: Disease-specific survival
