# Supplementary material for: Angiotensin receptor blocker and angiotensin-converting enzyme inhibitor use and survival in gastric cancer patients: a Finnish nationwide cohort study
Source: Gastric Cancer. 2025 Sep 20;28(6):1058–66. doi: 10.1007/s10120-025-01662-2 (PMC12630236; doi:10.1007/s10120-025-01662-2)
Supplement: Supplementary file 1 — Supplementary file1 (PDF 222 kb) [file 10120_2025_1662_MOESM1_ESM.pdf]

Supplement Table S1. Drugs and their ATC codes used in the study.

Drug category	Drug name	ATC codes
Angiotensin receptor blockers (ARBs)	Eprosartan	C09CA02
	Candesartan	C09CA06, C09DA06
	Losartan	C09CA01, C09DA01
	Olmesartan medoxomil	C09CA08, C09DA08
	Telmisartan	C09CA07, C09DA07
	Valsartan	C09CA03, C09DB01, C09DA03
Angiotensin-converting enzyme inhibitors (ACEIs)	Enalapril	C09AA02, C09BA02, C09BB02
	Lisinopril	C09AA03, C09BA03
	Perindopril	C09AA04, C09BA04, C09BB04
	Ramipril	C09AA05, C09BA05

Supplement Table S2. Comorbidities by drug use.

	Overall		Non-user		ARB user		ACEI user		
	n	(%)	*n*	(%)	*n*	(%)	*n*	(%)	p^a
Myocardial infarction									<0.001
No	2090	(93)	1597	(94)	265	(94)	228	(86)	
Yes	156	(6.9)	101	(5.9)	17	(6.0)	38	(14)	
Congestive heart failure									0.006
No	2092	(93)	1588	(94)	268	(95)	236	(89)	
Yes	154	(6.9)	110	(6.5)	14	(5.0)	30	(11)	
Peripheral vascular disease									0.009
No	2103	(94)	1593	(94)	271	(96)	239	(90)	
Yes	143	(6.4)	105	(6.2)	11	(3.9)	27	(10)	
Cerebrovascular disease									0.009
No	1992	(89)	1519	(89)	252	(89)	221	(83)	
Yes	254	(11)	179	(11)	30	(11)	45	(17)	
Dementia									0.030
No	1953	(87)	1491	(88)	244	(87)	218	(82)	
Yes	293	(13)	207	(12)	38	(13)	48	(18)	
Chronic pulmonary disease									0.2
No	1954	(87)	1488	(88)	237	(84)	229	(86)	
Yes	292	(13)	210	(12)	45	(16)	37	(14)	
Rheumatologic disease									0.12
No	2185	(97)	1654	(97)	277	(98)	254	(95)	
Yes	61	(2.7)	44	(2.6)	5	(1.8)	12	(4.5)	
Peptic ulcer disease									0.70
No	1885	(84)	1442	(85)	226	(80)	217	(82)	
Yes	361	(16)	256	(15)	56	(20)	49	(18)	
Mild liver disease									0.9
No	2216	(99)	1676	(99)	278	(99)	262	(98)	
Yes	30	(1.3)	22	(1.3)	4	(1.4)	4	(1.5)	
Diabetes without chronic complication									<0.001
No	1992	(89)	1542	(91)	237	(84)	213	(80)	
Yes	254	(11)	156	(9.2)	45	(16)	53	(20)	
Diabetes with chronic complication									0.7
No	2181	(97)	1652	(97)	272	(96)	257	(97)	
Yes	65	(2.9)	46	(2.7)	10	(3.5)	9	(3.4)	
Hemiplegia or paraplegia									0.2
No	2237	(100)	1693	(100)	280	(99)	264	(99)	
Yes	9	(0.4)	5	(0.3)	2	(0.7)	2	(0.8)	
Renal disease									0.4
No	2211	(98)	1668	(98)	279	(99)	264	(99)	
Yes	35	(1.6)	30	(1.8)	3	(1.1)	2	(0.8)	
Any malignancy									0.012
No	1964	(87)	1485	(87)	235	(83)	244	(92)	
Yes	282	(13)	213	(13)	47	(17)	22	(8.3)	
Moderate or severe liver disease									>0.9
No	2241	(100)	1693	(100)	282	(100)	266	(100)	
Yes	5	(0.2)	5	(0.3)	0	(0)	0	(0)	
Metastatic solid tumor									0.8
No	2240	(100)	1693	(100)	281	(100)	266	(100)	
Yes	6	(0.3)	5	(0.3)	0	(0.4)	0	(0)	
AIDS/HIV									>0.9
No	2246	(100)	1698	(100)	282	(100)	266	(100)	
Yes	0	(0)	0	(0)	0	(0)	0	(0)	

[a]Pearson's Chi-squared test, when appropriate Fisher's exact test.
ARB, angiotensin receptor blocker; ACEI, angiotensin converting enzyme inhibitor.

Angiotensin receptor blocker and angiotensin converting enzyme inhibitor use and survival in gastric cancer patients: A Finnish nationwide cohort study

Gastric Cancer

Auvinen A, Aaltonen P, Mustonen H, Caj Haglund, Puolakkainen P, Seppänen H

Department of Surgery, Translational Cancer Medicine Research Program, iCAN Digital Precision Cancer Medicine Flagship, Faculty of Medicine, University of Helsinki and Helsinki University Hospital, Helsinki, Finland.

Correspondence aliisa.auvinen@helsinki.fi
